# NGF-mediated transcriptional targets of p53 in PC12 neuronal differentiation

**DOI:** 10.1186/1471-2164-8-139

**Published:** 2007-05-31

**Authors:** Christopher Brynczka, Paul Labhart, B Alex Merrick

**Affiliations:** 1 National Institute of Environmental Health Sciences, National Institutes of Health, Research Triangle Park, North Carolina 27709, USA; 2 Department of Environmental and Molecular Toxicology, North Carolina State University, Raleigh, North Carolina 27606, USA; 3 Genpathway, Inc., San Diego, California 92121, USA

## Abstract

**Background:**

p53 is recognized as a critical regulator of the cell cycle and apoptosis. Mounting evidence also suggests a role for p53 in differentiation of cells including neuronal precursors. We studied the transcriptional role of p53 during nerve growth factor-induced differentiation of the PC12 line into neuron-like cells. We hypothesized that p53 contributed to PC12 differentiation through the regulation of gene targets distinct from its known transcriptional targets for apoptosis or DNA repair.

**Results:**

Using a genome-wide chromatin immunoprecipitation cloning technique, we identified and validated 14 novel p53-regulated genes following NGF treatment. The data show p53 protein was transcriptionally activated and contributed to NGF-mediated neurite outgrowth during differentiation of PC12 cells. Furthermore, we describe stimulus-specific regulation of a subset of these target genes by p53. The most salient differentiation-relevant target genes included *wnt7b *involved in dendritic extension and the *tfcp2l4/grhl3 *grainyhead homolog implicated in ectodermal development. Additional targets included *brk*, *sdk2*, *sesn3*, *txnl2*, *dusp5*, *pon3*, *lect1*, *pkcbpb15 *and other genes.

**Conclusion:**

Within the PC12 neuronal context, putative p53-occupied genomic loci spanned the entire *Rattus norvegicus *genome upon NGF treatment. We conclude that receptor-mediated p53 transcriptional activity is involved in PC12 differentiation and may suggest a contributory role for p53 in neuronal development.

## Background

The tumor suppressor p53 is recognized as a critical regulator of cell cycle progression and apoptosis, incorporating signals from DNA damage and other cellular stressors to decide cell fate [1]. Disruption of p53 activity through direct mutation or regulatory dysfunction is a demonstrated causal factor in a large proportion of human malignancies [2]. As a transcription factor, many of the functional cellular roles of the p53 protein are elicited through direct DNA binding to sequence-specific cis-regulatory elements [3] leading to the transactivation or transrepression of target genes. Many of these target genes have been described through both computational and biochemical methods [4-7].

Evidence suggests a functional role for p53 in differentiation and development, particularly within the nervous system. While initial p53-null mouse models were described as phenotypically normal at birth with markedly increased tumor development later in life [8,9], later studies demonstrated a proportion of p53^-/- ^mice had defects in neural tube closure resulting in exencephaly [10,11]. *In vivo *measurements in p53-responsive *lacZ *transgenic mice demonstrated elevated p53 transcriptional activity in the embryonic and early gestational nervous system [12], while high levels of p53 expression have been described throughout the chick, rat, mouse and *Xenopus *embryo [13-17] through midgestation. p53 functional activity has been further implicated in the differentiation of neuronal precursors [10,11,16] and is transcriptionally active within the nucleus of differentiating primary hippocampal neurons and oligodendrocytes [18], while p53 is inactive and localized to the cytoplasm of postmitotic differentiated sympathetic neurons [19]. p53 transcriptional activity may be further involved in the differentiation of other non-neuronal cell types. Transcriptional activity of p53 plays a direct role in embryonic stem cell differentiation by suppression of the *Nanog *gene [20], and can directly induce *Xenopus *homebox gene expression [21]. Involvement of p53 has also been shown in spermatogenesis [22], eye development [23] renal development [24], osteogenesis [25], immune development [26], lung development [27] and muscle differentiation [28]. Despite data supporting the transcriptional activity of p53 protein in differentiation, few genetic targets have been described and fewer still within a neuronal context.

Discovery of non-genotoxic mechanisms through which p53 protein may be activated within tumor cells harboring wild-type p53 remains an important therapeutic objective, both in effort to alleviate traditional off-target chemotherapeutic side-effects and to prevent the unpredictable mutagenicity characteristic of these therapies. Wild-type p53 can be activated in several malignancies by treatment with nerve growth factor [29] or pharmacologic agents [30-33], occurring through mechanisms sparing cells from the concomitant genotoxicity following chemotherapy. Recent evidence demonstrates that while p53 transcriptional regulation of target genes may be stimulus-dependent (e.g. DNA damage), promoter occupancy is generalized and may occur independently of the activating genotoxic stimulus [34]. Whether this paradigm is also applicable to nongenotoxic p53 stimuli compared to DNA damage-induced p53 activation is unclear. NGF-mediated p53 activation mainly results in decreased proliferative ability of tumor cells and not apoptosis [29], raising the question of whether NGF-regulated p53 activity is distinguishable from other means of p53 activation like DNA damage. While the mechanism of NGF-induced p53 activation is uncertain, inquiry into the transcriptional program following such activation is warranted to discern possible receptor-dependent p53 transactivational selectivity compared to genotoxic agents.

In the current study, the p53 transcriptional program was studied during nerve growth factor-induced differentiation of ectoderm-derived rat PC12 pheochromocytoma cells. A global chromatin immunoprecipitation-based screen was used to identify genetic elements bound to and regulated by p53. Treatment of PC12 cells with NGF induces differentiation into neuron-like cells marked by cell cycle arrest and neurite extension [35] along with marked changes in gene expression [36] and signaling pathway activity [37]. NGF-responsive p53 activity [38] has a functional contribution towards NGF-mediated PC12 differentiation [39-41], but a role in gene-specific transactivation has not yet been established. We hypothesized that marked accumulation of p53 protein within differentiating PC12 cells would be accompanied by concomitant transcriptional regulation of genes involved in differentiation and cell cycle arrest. Furthermore, we hypothesized that genomic elements regulated by p53 during neuronal differentiation may be unique from those regulated during genotoxic stress and apoptosis. We now report that p53 protein is transcriptionally activated in NGF-mediated neuronal differentiation and describe its binding to a number of novel and previously unreported genomic regions as well as the transcriptional outcome of this binding. We identify and validate genomic targets of p53 activity within the differentiating neuron-like cell, and demonstrate selectivity between NGF-activated versus genotoxicant-activated p53 transcriptional activity in a select number of p53 target genes.

## Results

### NGF induces activation of p53 protein within PC12 cells

**Figure 1:**
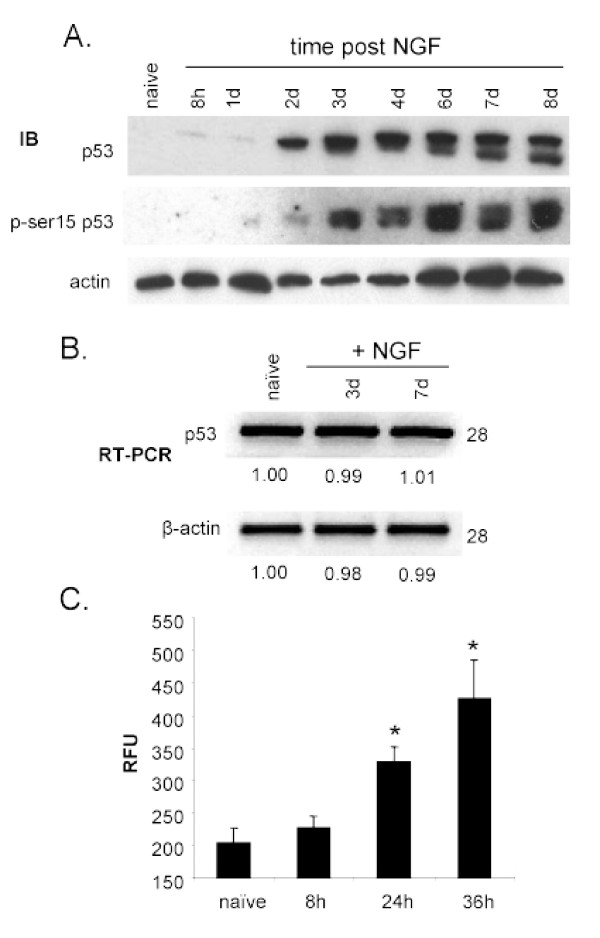
PC12 neuronal differentiation involves p53 stabilization and activation. A) Immunoblot depicting total and phospho-serine 15 p53 protein levels over time following 50 ng/mL NGF 2.5S, with total actin protein levels serving as loading control. B) RT-PCR levels of p53 mRNA over time following NGF 2.5S treatment compared to β-actin loading control. C) Luciferase activity in PC12 cells transfected with p53TAluc reporter vector following NGF 2.5S treatment at indicated time intervals, where p ≤ 0.05. Error bars represent standard deviation.

**Figure 2:**
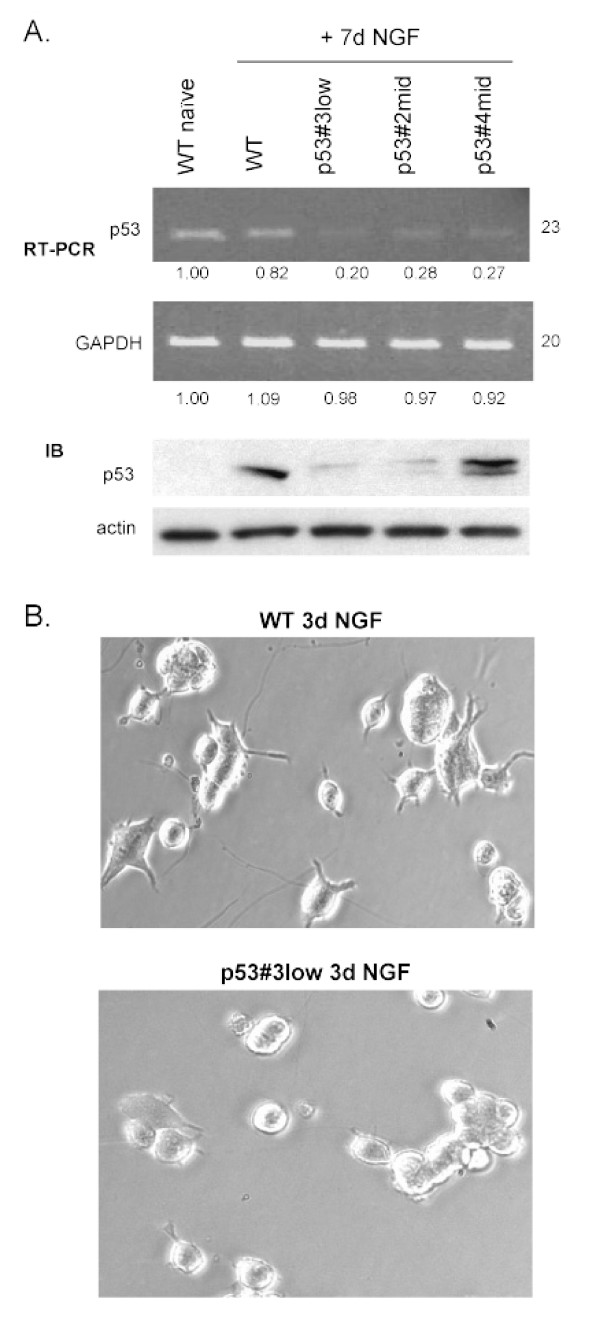
Analysis of p53 knockdown PC12 cell lines. A) RT-PCR and immunoblot depicting levels of p53 mRNA and protein in wild-type naïve PC12 cells compared to 7-day NGF (50 ng/mL) differentiated wild-type and anti-p53 shRNA expressing PC12 cells. GAPDH mRNA levels serve as loading control for RT-PCR, while actin serves as immunoblot loading control. p53 and GAPDH mRNA levels in each sample were quantitated using ImageJ and normalized to naïve RNA levels, depicted under each band. PCR cycle number used is shown on right of gel image. p53 RNA and protein levels (and knockdown efficiency) were lowest in p53#3low cells, followed by p53#2mid and p53#4mid cell lines. B) Representative image of wild-type and p53#3low PC12 cells following 3 days treatment with 50 ng/mL NGF.

PC12 morphologic changes and neurite extension consistent with differentiation were visible within 12 hours of NGF addition to the media and became marked over the course of 7 days [See Additional file 1; Figure 1A]. NGF-dependent differentiation after 7 days treatment in PC12 cells was evidenced by expression of the dopamine transporter, a marker for dopamine generation [42] and transport, and decreased nestin immunoreactivity, indicating loss of undifferentiated phenotype [43] [See Additional file 1; Figure 1B]. In agreement with previous reports [44], cell cycle analysis by flow cytometry using FITC-BrdU labeling of total and newly synthesized DNA, respectively, revealed the population of cells within S phase was decreased to ~1% of the total cell population within 96 hours of NGF treatment [See Additional file 1; Figure 2A]. Reduction of S phase cells was accompanied by a concomitant increase in the number of cells within G1 phase after 7 days of NGF exposure [See Additional file 1; Figure 2B].

p53 protein levels were significantly elevated in response to NGF treatment in PC12 cells as previously described by others [44,45]. Accumulation of p53 protein was detectable at 8 hours following NGF exposure and remained highly elevated over the course of 7 days (Figure 1A). Phosphorylation of p53 protein at serine 15, which is typically needed for transactivation, began at about 8 hours after NGF treatment and remained highly phosphorylated throughout 7 days (Figure 1A). Accumulation of p53 protein can be attributed to protein stabilization since levels of p53 mRNA remained unchanged over a 7-day course of NGF treatment (Figure 1B). Virtually all p53 protein was localized within the nucleus in 7 day NGF-differentiated cells, as determined by immunoblot of nuclear and cytoplasmic fractions (data not shown). Accumulated p53 protein was transcriptionally active, as demonstrated by luciferase expression using the p53TA-luc reporter vector in differentiated compared to naïve cells (Figure 1C).

The role of p53 activity in differentiation of NGF treated PC12 cells was investigated in anti-p53 shRNA-expressing PC12 cells (Figure 2A). Three stable PC12 lines expressing different anti-p53 shRNAs were created to observe p53-dependent function in PC12 differentiation. p53 RNA silencing resulted in reduced p53 protein levels, with p53#3low having the lowest levels, followed by p53#2mid and p53#4mid. The p53#4mid cell line reproducibly expressed p53 protein levels approaching those observed in the wild-type cell. In combination, these three shRNA cell lines formed a gradient of p53 protein expression compared to the wild-type cell. While no apparent morphological differences were noted in the shRNA expressing cell lines maintained in the naïve state, NGF differentiation of these cells demonstrated reduced early neurite extension compared to their wild-type counterparts. Within 3 days of NGF exposure, p53#3low cells had markedly reduced neurite outgrowth compared to wild-type cells (Figure 2B), lasting up to 7 days (data not shown). These results agree with prior work showing disruption of morphological differentiation following both dominant-negative and temperature-sensitive p53 transfection into PC12 cells [18,39,46].

### Chromatin immunoprecipitation and cloning of ChIP-generated tags

**Figure 3:**
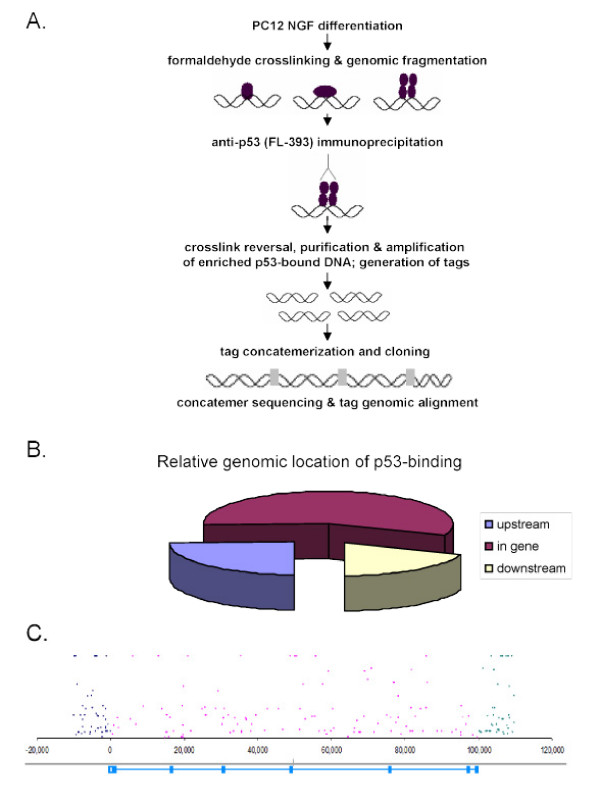
Identification of p53-occupied genomic sites. A) Schematic of chromatin immunoprecipitation (anti-p53, FL-393) cloning strategy. See methods for more detail. B) Location of tags containing putative p53 binding sites relative to known genes within the rat genome. Binding sites scoring 90 or above out of 100 using the p53MH algorithm were included in this analysis. C) A hypothetical 100 kb gene map is used to show the relative location of 364 of experimentally determined p53 site-containing tags that achieved scores of ≥ 90 with the p53MH predictive binding algorithm. Data show that p53 site-containing tags were found in the upstream 5', downstream 3' and intergenic gene regions.

p53 DNA binding was studied in **s**even-day differentiated PC12 cells via chromatin immunoprecipitation (ChIP) analysis. Preliminary assay validation demonstrated p53 binding to a known, well-defined binding site within the *p21*^*Waf*1/*Cip*1 ^promoter at a 2-fold greater level in differentiated versus naïve cells [See Additional file 1; Figure 3]. Greater than 10-fold increased p53 occupancy at the p21^Waf1/Cip1 ^proximal binding site was detected with FL-393 immunoprecipitating antibody compared to IgG alone, confirming antibody specificity [See Additional file 1; Figure 3]. Having validated p53 occupancy to a known gene promoter, ChIP-enriched DNA from differentiated PC12 cells was amplified, concatemerized and cloned. Cloned concatemers were sequenced and separated, resulting in 7,184 individual DNA tags representative of putative p53-occupied locations within the PC12 genome [See Additional file 1; Table 1]. Tags were aligned to the *Rattus norvegicus *genome and locations with >1 tag mapping within a 2 kb area ("clusters") were identified. Cluster chromosomal coordinates and sequences have been compiled and organized [see Additional File 2]. Sequence data were obtained for 500 bp upstream and downstream of each tag, single or clustered, and tags were recompiled to represent the original p53-bound ChIP-enriched fragments. Since the number of sequenced tags were non-saturating compared to the total population of p53-occupied fragments, both clusters and single tags were potentially informative. Tags representing putative p53-occupied genomic fragments were found to span the entire *Rattus norvegicus *genome in addition to known p53 binding regions within the *MDM2*, *cyclin G1 *and other known target promoters, validating this experimental approach for the study of other novel putative targets.

Recompiled tag sequences were analyzed for predicted p53 binding sites using the p53MH algorithm [47] and sorted according to both binding site score and location relative to nearby genetic elements. Stringency for p53 binding site probability score was set at a cutoff of 90 (out of 100) as determined by the p53MH algorithm using likelihood ratio scoring and a 14 base gap size limit between half sites. Results using these criteria demonstrated 364 p53 binding sites within tag sequences located in upstream 5', downstream 3' and intergenic gene regions. Of those tags with a p53MH binding site score ≥ 90 (out of 100), 56% mapped to within a gene, 25% to the upstream 5' region and 19% to the downstream 3' region (Figure 3B). Observed p53-occupied sites in the PC12 genome were visualized by plotting normalized tag alignment locations relative to a hypothetical gene size of 100 kb (Figure 3C). Scored p53 binding sites clustered to both the 5' and 3' gene regions, while intergenic binding sites were observed throughout the entire length of the gene.

### Validation of p53 occupancy and transcriptional regulation

**Figure 4:**
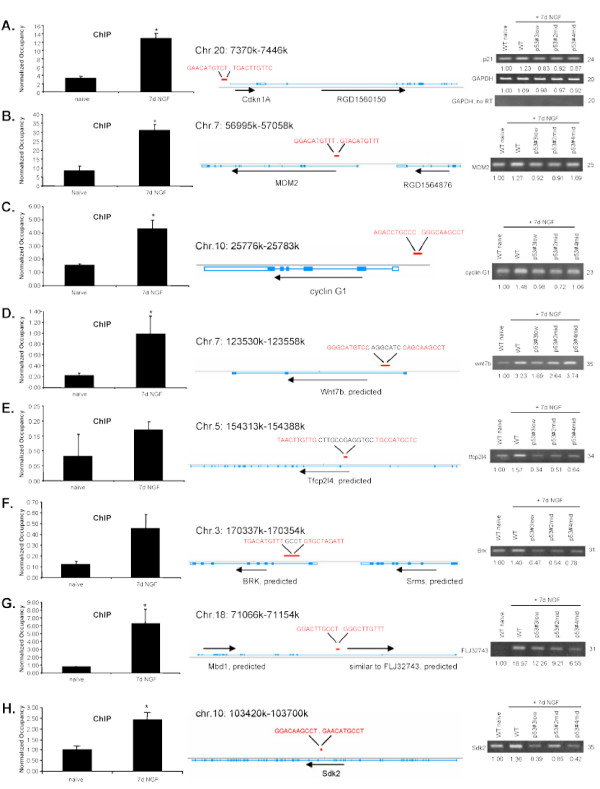
p53-dependent induction of target genes. Panels A-H show site-specific qPCR ChIP results on left with standard deviation, where significance at p ≤ 0.05 is designated by an asterisk (*). Center tile depicts gene map of predicted p53 binding site location and the sequence relative to annotated genes, along with exon structure and direction of transcription as per NCBI MapViewer conventions. Right tile represents RT-PCR gel image of each mapped target gene. RT-PCR analyses were performed multiple times for each target gene and gel images are representative of consistent expression levels. mRNA levels of each gene in wild-type naïve PC12 cells were compared to 7-day NGF (50 ng/mL) differentiated wild-type and three anti-p53 shRNA expressing PC12 cells (p53#3low, p53#2mid, p53#4mid). ImageJ quantitation, normalized to naïve RNA levels, is depicted under each band, while PCR cycle number is depicted on right of gel image. GAPDH expression levels are shown in A and represent a typical loading control along with no-RT negative control.

**Figure 5:**
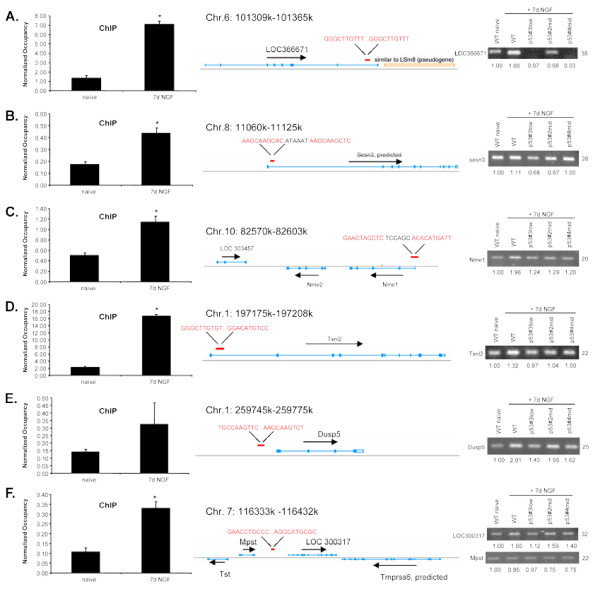
p53-dependent induction of additional target genes. Panels A-F show site-specific qPCR ChIP results on left with standard deviation, where significance at p ≤ 0.05 is designated by an asterisk (*). GAPDH controls for RT-PCR in Figure 4 also apply to gene expression analyses in Figure 5. Note the p53-occupied sequence in Panel F lies between genes which were both examined for changes in expression. Overall display and analyses are as described in Figure 4 legend.

**Figure 6:**
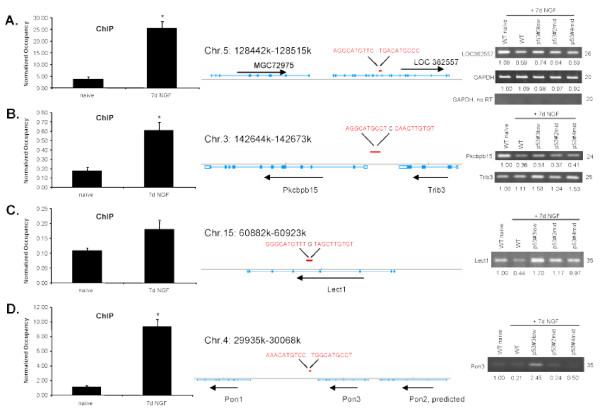
p53-repressed target genes. Panels A-D show site-specific qPCR ChIP results on left with standard deviation, where significance at p ≤ 0.05 is designated by an asterisk (*). Note the p53-occupied sequence in Panel B lies between genes which were both examined for changes in expression. Display and analyses are as described in Figure 4 legend.

**Figure 7:**
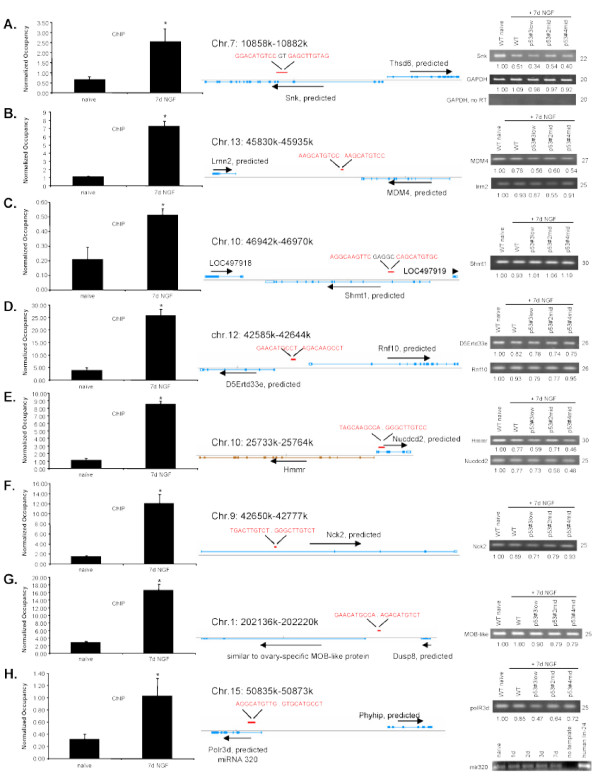
Targets with NGF-dependent increase in p53 occupancy, but where p53-dependent transactivation could not be clearly determined using anti-p53 shRNA expressing cell lines. Panels A-H show site-specific qPCR ChIP results on left with standard deviation, where significance at p ≤ 0.05 is designated by an asterisk (*). Note the p53-occupied sequences in Panels B, D and E lie between two genes so that were both examined for changes in expression. Display and analyses are as described in Figure 4 legend.

**Figure 8:**
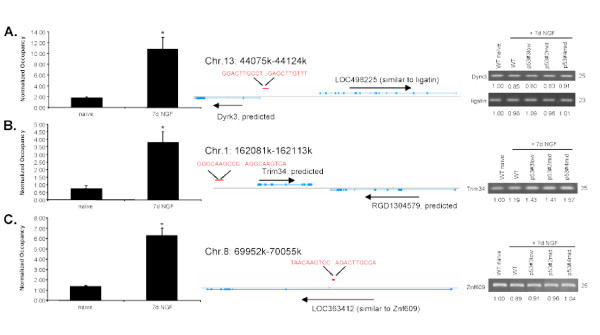
Additional targets with NGF-dependent increase in p53 occupancy, but where p53-dependent transactivation could not be clearly determined using anti-p53 shRNA expressing cell lines. Panels A-C show site-specific qPCR ChIP results on left with standard deviation, where significance at p ≤ 0.05 is designated by an asterisk (*). GAPDH controls in Figure 7 also apply to gene expression analyses in Figure 8. Note the p53-occupied sequence in Panel A lies between genes which were both examined for changes in expression. Display and analyses are as described in Figure 4 legend.

We selected a total of 100 clusters and single tags combined for ChIP-based validation of p53 occupancy based on either the presence of a putative p53 binding site as determined by the p53MH algorithm, or tag location relative to a genomic element of interest. Since tag sequencing and identification were not performed to saturation we considered individual tags and clusters as potentially informative. We recognized that selection based on high p53 binding scores alone may pose a constraint on putative regulated genes because p53-mediated transrepression can occur in both the absence of a consensus binding site [48,49] or by binding to sites sufficiently distinct from the consensus sequence that fall below our accepted p53MH binding site prediction level [50]. A preponderance of genes whose known functions may be relevant in neuronal differentiation also factored into our selection of tags and clusters for validation. We selected 100 tags for ChIP-based validation based on criteria of ontological classification (i.e. GO terms related to development, differentiation or morphogenesis), known function of associated genes, or p53 binding site score in order to identify candidate p53-regulated targets with a role in PC12 differentiation. ChIP analysis demonstrated significantly elevated (p ≤ 0.05) p53 occupancy in an NGF-dependent manner for roughly 30% of the 100 studied validation targets. Those targets to which p53 binding was significantly increased in NGF-treated cells are shown in Figures 4, 5, 6, 7, 8. As expected, reproducible binding was demonstrated to promoters of known p53-inducible target genes shown in Figures 4 and 5 including p21^Waf1/Cip1^, MDM2 and cyclin G1 (Figure 4). In addition to NGF-responsive genomic p53 binding sites, roughly 40% of targets showed significant p53-binding independent of NGF treatment [See Additional file 1; Table 3]. These targets were significantly enriched in ChIP validation when compared to negative control regions, although no NGF-responsive changes were noted in p53 binding. Absence of NGF-dependent 53 binding to these regions precluded further analysis since they were beyond the objectives of this study. While these genomic locations were ChIP-enriched, cloned and demonstrated to be enriched once more in a validation ChIP experiment, further study will be necessary to demonstrate p53-dependent transcription.

In order to determine the transcriptional outcome of p53 binding to NGF-responsive target regions described above, RT-PCR was performed to compare mRNA levels for indicated targets among naïve and differentiated PC12 cells (Figures 4, 5, 6, 7, 8). p53-dependence on target gene expression was verified using three stable anti-p53 shRNA-expressing PC12 lines with varying p53 knockdown efficiencies. Use of the three shRNA clones formed a gradient of p53 mRNA and protein expression (Figure 2A), where p53#3low had the lowest level of expression and p53#2mid & p53#4mid were both reproducibly higher with a moderate level of p53 expression. Including known targets, a total of 14 genes were p53-induced in differentiating PC12 cells. Importantly, we discovered ten novel, p53-inducible targets regulated in an NGF-responsive manner (Figures 4 and 5), including the *sesn3 *and *nme1 *targets recently described to be p53 regulated either by association or indirect experimental evidence [51,52]. In addition, the *dusp5 *gene was an NGF-inducible p53 target, recapitulating earlier reports of this gene being p53-regulated [53]. Further, we report four novel p53 gene targets that demonstrated a p53-dependent repression (Figure 6). These included the *pkcbpb15/trib3 *locus, where transcripts of both genes were elevated in p53-silenced cells. We also demonstrate eleven p53 occupied regions where transcriptional changes in nearby genes during NGF-mediated differentiation could not be accredited solely to p53 activity in the study (Figures 7 and 8), including the previously described p53 targets *snk *[54] and *nck2 *[5]. Additionally, new p53-occupied binding sites were identified for the *MDM4 *and *dyrk3 *genes, for which different p53 binding locations have previously been described [5].

**Figure 9:**
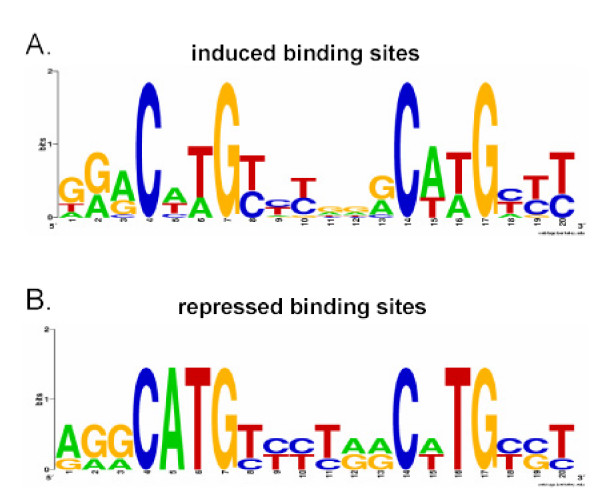
WebLogo multiple sequence alignment of p53 binding sites. Individual base letter height indicates level of conservation within each binding site position. A) Consensus sequence of p53-induced targets shown in Figures 4 and 5. B) Consensus sequence of p53-repressed targets shown in Figure 6.

The majority of p53MH-predicted p53 binding sites within regions of validated occupancy contained either no spacer or a short spacer, in agreement with earlier data [5]. However, there are several notable exceptions to this trend including *wnt7b*, *grhl3*, *shmt1 *and *sesn3 *binding sites, which contain atypical spacers from 5–13 bp separating the individual half-sites, similar to the described p53-binding site within the *siah *promoter [55]. Consensus binding sites derived from our data are shown in Figure 9 using the WebLogo [56] sequence analysis tool. We report little deviation from the p53 consensus sequence for transactivation (Figure 9a) compared to repression except for the substitution of purine for pyrimidine at site 11 (Figure 9b). This data supports the capability for p53-regulated transcriptional repression from a consensus binding site [57].

### Stimulus-dependent transcriptional regulation

**Figure 10:**
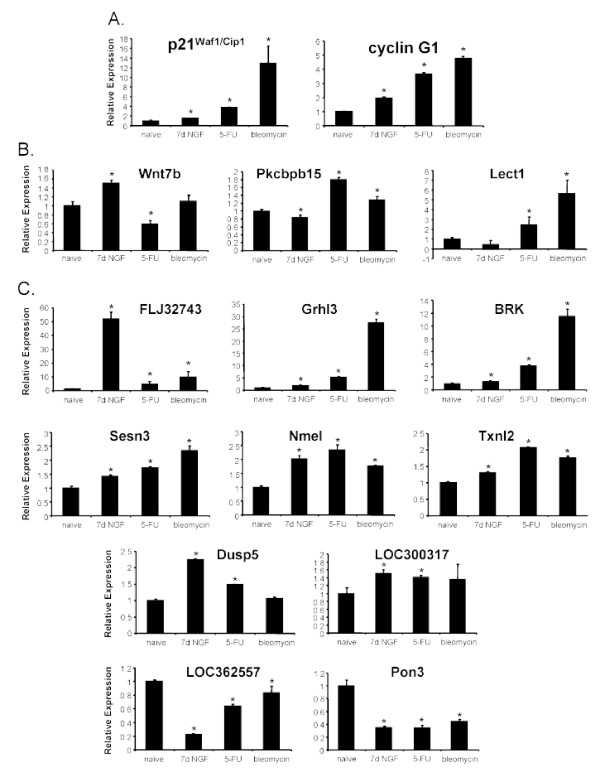
qRT-PCR expression analyses of p53 targets following genotoxic exposure. A) mRNA expression levels of known p53 targets comparing naïve, 7-day NGF (50 ng/mL), 6 h 5-fluorouracil (375 μM) and 6 h bleomycin (10 μg/mL) exposure. Expression was measured via real time PCR and normalized to naïve expression levels using 2^-ΔΔct ^where significance at p ≤ 0.05 is designated by an asterisk (*). Error bars represent standard deviation. B) Same as in Panel A, depicting genes whose expression levels were not regulated in the same manner between NGF treatment and genotoxic treatment. C) Same as in Panel A, depicting genes with comparable regulation between NGF treatment and genotoxic exposure.

Experiments were conducted to determine if p53-regulated genes in differentiation were unique from those following genotoxicity. Expression levels of NGF-dependent p53 target genes measured after DNA-damage by bleomycin and 5-fluorouracil were compared to NGF treatment to discriminate stimulus-dependency of p53-mediated transcription. As expected, expression levels for *p21*^*Waf*1/*Cip*1 ^and *cyclin G1 *were elevated following all treatments, confirming the transcriptional activity of p53 in all groups (Figure 10A). Increased expression levels in three of the NGF-responsive targets, *wnt7b*, *pkcbpb15 *and *lect1 *(Figure 10B) were not recapitulated in 5-FU and bleomycin treated cells. Results demonstrate an opposite transcriptional regulation for these p53-regulated targets depending upon the treatment stimulus. Although the absolute levels varied, expression levels and direction of the remaining p53-regulated target genes were similar regardless of the treatment (Figure 10C). Expression levels of all gene targets were similar to those found earlier using RT-PCR, with absolute differences attributed mainly to differential assay sensitivities. These data demonstrate that the gene expression at chosen time points for some, but not all, p53 target genes is NGF-dependent. The remaining target genes studied, however, were induced or repressed both by NGF receptor-mediated or genotoxic p53-inducing stimuli.

## Discussion

The p53 tumor suppressor is widely appreciated as a central node in the regulation of proliferation and apoptosis in response to various genotoxic insults. Its role within cellular differentiation, however, is uncertain. p53 has been considered non-essential to development since p53 null mice are viable and succumb to tumors rather than developmental malformations [8]. The discovery of low frequency neural tube defects in p53-deficient mice [11] is an apparent paradox, suggesting the role of p53 in development may be more complex than initially believed. Interpretation of developmental effects in p53-deficient models at the organismal level are complicated by the overlapping transcriptional functions of the p53 family members p63 and p73 [58] which include such vital targets as p21^Waf1/Cip1^, Bax and MDM2 and likely other unknown target genes. In addition, regulation of growth control and apoptosis can also be carried out through p53-independent cellular mechanisms which could circumvent developmental defects in the majority of p53-deficient mice.

The marked accumulation of p53 protein and serine 15 phosphorylation levels suggesting its activation during NGF exposure led us to investigate a possible transactivational role for p53 in neuronal differentiation. Using the PC12 cell model of NGF-induced neuronal differentiation, we hypothesized that p53 is involved in differentiation through specific transcriptional regulation of target genes. A contribution of p53 to PC12 cell differentiation is suggested by the reduction of neurite outgrowth observed during p53 knockdown studies described here, which is consistent with previous work showing similar findings using a temperature-sensitive p53 mutant [39,40]. However, we demonstrate p53-dependence for target gene transcription using three PC12 cell lines with varying levels of shRNA-mediated p53 silencing, further enabling inference into the transcriptional sensitivity of each target gene to reduced levels of p53 protein. The 7-day time point of NGF treatment was selected for further analysis to explore p53 transcriptional activity at the time of highest p53 protein accumulation representing a stable timeframe of p53-DNA occupancy following NGF exposure. As the ChIP-cloning approach utilized here is essentially a snapshot of p53-DNA occupancy following 7 days of NGF exposure, it is interesting to consider that the population of p53-regulated gene targets may vary over time following NGF treatment.

Here we describe a number of novel p53-occupied genomic sites within the NGF-differentiated *Rattus norvegicus *neuronal PC12 cell line, and further identify corresponding genes regulated by p53 binding to these regions. The transcriptional targets described here represent enticing mechanisms through which PC12 cellular differentiation may be controlled by activated p53. Particularly interesting is the regulation of *wnt7b *and the *tfcp2l4 *grainyhead homolog gene. Transcriptional regulation of Wnt pathway genes by p53 has been described for several factors, including the negative regulator Dickkopf-1 [59], the transcription factor Tcf-4 [60] and beta-catenin [61]. Until now, direct DNA binding and transcriptional regulation of Wnt genes themselves has not been previously reported for p53. Wnt7b functional activity is necessary for dendritic development and neuronal connectivity in hippocampal neurons [62], consistent with the generalized lack of neurite extension observed in NGF-treated p53 knockdown PC12 cells in this study. The transcriptional regulation of *wnt7b *by p53 may be extensible to other cell types. For example, macrophage-derived wnt7b has been reported as an apoptotic initiator in vascular endothelial cells of the developing eye [63], raising the possibility of p53 apoptotic control in immune cells through localized secreted factors.

The *transcription factor cp2-like 4 *gene, a *Rattus norvegicus *grainyhead family homolog, is homologous to the *grhl3 *mouse and human family members. In *Drosophila*, the grainyhead gene product participates in wnt-frizzled signaling in the establishment of wing planar polarity [64] and in cuticle barrier formation and function [65]. The latter function is conserved in mammals, where grainyhead homologs are involved in epidermal stratification and wound repair [66]. The importance of the grhl3 transcription factor during development is underscored in the *grhl3*-null mouse, in which neural tube defects are prevalent [67,68]. Absence of the p53-*tfcp2l4 *network in p53-null mice may represent a potential mechanism through which a proportion of these mice are born with neural tube defects. The role of grhl3 in ectodermal differentiation and specification into epidermal tissue and also within epidermal wound healing is strikingly similar to the demonstrated involvement of the p53 family member p63 in these events [69]. Since p63 has an overlapping transcriptional program with p53 [58], investigation into joint regulation of *tfcp2l4 *is warranted as a possible common juncture of *in vivo *redundant transcriptional control in development.

Other transcriptional targets of p53 found in our study may play roles within the differentiating PC12 cell or developing organism. The *nme1/nm23 *gene is a positive regulator of neuronal differentiation through regulation of Rb2/p130 expression [70]. The Kctd (potassium channel tetramerization domain containing) family, of which *FLJ12242 *is a member, is involved in the left-right symmetry of developing zebrafish brain [71]. We found *FLJ12242 *to be the most highly expressed p53-regulated gene in NGF-mediated differentiation. *Txnl2 *is involved in P19 cardiac cell differentiation [72], while *BRK *promotes enterocyte differentiation [73]. The *Sdk2 *neuronal adhesion molecule is involved in synaptic connectivity [74]. *Dusp5 *is a negative regulator of ERK signaling [75], thus regulating MAPK signal propagation. The *Lect1/Chm1 *gene, an anti-angiogenic factor in differentiating cartilage [76] and a bone remodeling factor [77], may be involved in bone development. Furthermore, the additional unannotated p53-regulated targets described here such as *FLJ32743*, *LOC366671 *and *LOC362557 *may be important in yet unknown developmental, differentiation or signaling processes.

Data from previous studies have reported a number of p53 regulated genes that do not completely correspond to expression changes observed in PC12 differentiation. This observation suggests that p53 transcriptional regulation in response to diverse stimuli shows similar gene expression but also some level of stimulus specificity. For example, our data show an overlap of p53 occupancy at some genomic loci irrespective of the p53-activating stimulus when compared to results from a recent chromatin immunoprecipitation/paired-end ditag (ChIP PET) screen for p53 genomic occupancy in human colorectal cells treated with 5-fluorouracil, [5] and another study identifying p53-regulated genes through microarray analysis from irradiated wild-type and p53-null animals [54]. We confirmed the findings of p53 occupancy at the *Snk/Plk2 *[54], *MDMX*, *Nck2*, *Dyrk3, p21*^*Waf*1/*Cip*1^, *MDM2 *and *cyclin G1 *[5] regulatory regions but we observe p53-dependent expression changes only in the *p21*^*Waf*1/*Cip*1^, *MDM2 *and *cyclin G1 *targets with NGF treatment. Regulation of *Snk/Plk2*, *MDMX*, *Nck2 *and *Dyrk3 *may not completely depend upon p53 promoter binding alone but also on availability of other transcriptional cofactors. Since these targets were previously identified as p53-regulated, by extension, the genes we report as *shmt1*, *rnf10*, *hmmr*, *trim34*, *znf609 *and *ovary-specific MOB-like protein *may also be regulated under other p53-activating conditions. Along with the NGF-dependent regulation of p53 gene targets described in our study as *wnt7b*, *pkcbpb15 *and *lect*, we believe that the p53-occupied targets *shmt1, rnf10, hmmr, trim34, znf609 and ovary-specific MOB-like protein *may provide additional examples of stimulus-dependent p53 transcriptional targets. Interestingly, we did not find enrichment of p53-occupied loci in NGF-differentiated PC12 cells for mediators of apoptosis or DNA repair [see Additional file 2] probably because these recognized functions of p53 are not involved in NGF differentiation. For instance, we did not observe DNA repair genes, MLH1 and PMS2, found in a recent p53-binding genomic study through serial analysis of binding elements (SABE) in p53-expressing Jurkat cells and verified in cisplatin challenged fibroblasts [78]. In addition, computational approaches combining ChIP, DNA microarray with p53 sequence binding matrices have derived genomic maps of candidate p53 binding sites that reveal p53 regulation of a proapoptotic gene, BCL-G/BCL2L14 [79], various coding and non-coding genes [4] and regulation of micro-RNAs [80]. By comparison, we show that NGF-directed p53 transcription in neuronal cells regulated several genes unique to differentiation and different from transcriptional targets previously identified in other p53 genome-wide studies.

NGF receptor-mediated induction of p53 activity through TrkA [39,44] may represent a useful therapeutic avenue for development of non-genotoxic chemotherapeutics [29]. While activation of the p53 protein in response to NGF treatment within PC12 cells has recently been described [44], it is less clear what particular signaling proteins are responsible for the marked increase in p53 protein stability and transactivational selectivity. Changes in p53 activity are related, directly or indirectly, to TrkA activated signaling pathways, such as Ras/MAPK [81]. Activation of the low affinity NGF receptor p75^NTR ^by NGF results in apoptosis in Schwann cells [82] through the activity of p53 [83]. TrkA high affinity and p75^NTR ^low affinity NGF receptors may represent a useful model through which the divergent 'choices' between cell cycle arrest/differentiation and apoptosis by p53 protein function can be studied. Notably, NGF-induced PC12 cell differentiation results in little or no detectable apoptosis, despite markedly increased p53 levels and transcriptional activity over the extended time course of several days. It is thus likely that both the inducing agent (i.e. stimulus) and cell type lend specificity to the p53-controlled transcriptional response observed for other p53-responsive conditions [84]. In addition, the continued transcription of MDM2 and cyclin G1 described here is in apparent opposition to elevated p53 protein levels following NGF treatment. MDM2 functions as the major negative regulator of p53 protein within the cell [85], while cyclin G1 exerts its negative regulatory role through MDM2 dephosphorylation and subsequent stabilization [86]. However, continued stabilization of p53 protein during NGF-induced differentiation occurs despite the increased MDM2 and cyclin G1 RNA levels shown here and maintained MDM2 protein levels (data not shown). This suggests the presence of an intermediate event either inhibiting MDM2 function, or protecting p53 protein from MDM2 function, in differentiating PC12 cells. The incongruent time frames of activation for NGF-induced p53 compared to the relatively rapid stabilization via genotoxic agents further suggests the presence of a mechanism distinct from DNA damage through which p53 stabilization and activation may occur during NGF-mediated signaling.

The approach used here in the identification of p53-regulated elements in neuronal differentiation represents a useful strategy amenable to the analyses of DNA binding by other transcription factors. The recognized roles of NGF-induced p53 transcriptional targets identified in this report raise new prospects for a role of p53 in differentiation and development. Further, we provide evidence demonstrating stimulus-specific selectivity by activated p53 protein in transactivation.

## Conclusion

Nerve growth factor-mediated activation of p53-depedent transcription represents an unusual example of receptor-mediated, non-genotoxic p53 activation. Using a global chromatin immunoprecipitation cloning technique, we have identified and validated 14 novel transcriptional targets of NGF-activated p53 protein during neuronal PC12 differentiation. This study provides evidence of a functional role for p53 in differentiation.

## Methods

### Cell culture and treatments

PC12 cells (ATCC, Manassas, VA) were propagated in RPMI 1640 medium (Invitrogen, Carlsbad, CA) supplemented with 2 mM L-glutamine, 10% horse serum (Invitrogen), 5% fetal bovine serum (Invitrogen) and appropriate antibiotics in a humidified incubator maintained at 37° with 5% CO_2_. PC12 cells (passage 10–25) were differentiated on rat tail collagen-coated culture vessels (Sigma, St. Louis, MO) in RPMI 1640 medium containing 1% horse serum, 2 mM L-glutamine and antibiotics with 50 ng/mL mouse nerve growth factor, (NGF 2.5S) (Chemicon, Temecula, CA) for indicated time intervals. Differentiation medium was replaced and fresh NGF 2.5S was added every third day to differentiating cells. PC12 cells were incubated with genotoxic agents at the following concentrations, at the indicated time points: bleomycin, 10 μg/mL (Sigma) and 5-fluorouracil, 375 μM (Sigma). Reporter assay was performed by transient transfection of p53TA-luc vector (BD Pharmingen, San Diego, CA) into naïve PC12 cells plated onto collagen-coated dishes using Lipofectamine 2000 (Invitrogen). Cells were treated with NGF the following day and luciferase activity was quantified using the Luciferase Assay System (Promega, Madison, WI) at the indicated time intervals on a Tropix (Bedford, MA) TR717 luminometer. Statistical analysis of reporter assay data was performed using one-way ANOVA followed by Tukey's HSD post-hoc test for significance.

### Generation of stable shRNA expressing cells

Stable anti-p53 shRNA expressing PC12 cells were generated via lentiviral-mediated infection, integration and stable selection (BlockIt Lentiviral Expression System, Invitrogen). Hairpin sequences were designed and synthesized (IDT, Coralville, IA) with appropriate overhangs for cloning into the pENTR/U6 entry vector. The informative p53-targeting sense hairpin sequences are: p53-2mid – 5'-GCATACGGATTTCCTTCCACC-3', p53-3low – 5'-ATATCCGACTGTGAATCCTCC-3', p53-4mid – 5'-AAAATTAGGTGACCCTGTCGC-3'. shRNAs were designed using the 4-base loop sequence CGAA. Hairpin sequences were cloned into the pENTR/U6 vector and selected clones were sequence-verified. The U6 promoter-shRNA cassettes were transferred into the pLenti6-BLOCK-iT-DEST vector. The resulting destination clones were used to generate lentiviral particles by transfection of HEK293FT cells along with the pLP1, pLP2 and pLP/VSVG plasmids in equal amounts using FuGene (Roche Diagnostics, Indianapolis, IN) lipid reagent. Transfection efficiencies were monitored by concomitant tranfection of the pEYFP-C1 vector (BD Clontech, Mountain View, CA), demonstrating efficiency ≥ 90% in all cases. Lentiviral-laden media was collected 48 hours following transfection, clarified by centrifugation and used with 4 μg/mL polybrene (Sigma) in transduction of naïve PC12 cells plated on collagen. Selection using 6 μg/mL blasticidin was initiated and maintained for 7 days until stable lines were enriched. Cells were maintained for 14 days before differentiation and experimentation. Experiments using stable shRNA-expressing PC12 cells were compared to wild-type PC12 cells of the same original population and passage number in all cases.

### Western blotting

Sample preparation for SDS-PAGE was performed as previously described [87]. Antibodies used were against nestin (Rat401, Pharmingen), dopamine transporter (D2442, Sigma), p53 (pAb122, Pharmingen), phospho-serine 15 p53 (No. 9284, Cell Signaling, Davers, MA) and actin (MAb1501, Chemicon). Proteins were visualized using either ECL reagent (GEH Amersham, Piscataway, NJ) or SuperSignal reagent (Pierce, Rockford, IL).

### Chromatin immunoprecipitation assays

ChIP assays were performed similarly to previous reports [88,89]. Briefly, NGF-differentiated and naïve (dividing) PC12 cells were crosslinked by 1% formaldehyde with rocking for 15 minutes before terminating the crosslinking reaction with 125 mM glycine. Cells were washed, lysed by Dounce homogenization and resupended in a buffer containing sodium deoxycholate, SDS and Triton X-100 [88]. Chromatin was sheared to an average length of 500 bp by sonication on ice and samples were clarified by centrifugation at 10,000 × g. Sheared, crosslinked chromatin complexes were precleared with protein A agarose. p53-DNA complexes were immunoprecipitated using anti-full length p53 polyclonal antibody (FL-393, Santa Cruz Biotech, Santa Cruz, CA) or with control rabbit IgG (I-5006, Sigma). Immune complexes were captured with protein A agarose and beads were washed as described [88,89]. Protein/DNA complexes were eluted and crosslinks reversed by incubating at 65°C for at least 6 hours. To assay for p53 binding sites in purified ChIP DNA, target specific primers [See Additional file 1; Table 2] were used to measure amounts of target sequence in immunoprecipitated samples by qPCR using SYBR Green-based detection (BioRad, Richmond, CA). Experimental qPCR values were normalized against values obtained for 25 ng of input DNA using the same primer set. Non-specific enrichment of chromatin was minimal as determined by the amplification of enriched ChIP DNA using primers designed to multiple non-transcribed genomic regions.

### Cloning and analysis of enriched ChIP DNA

Cloning of enriched ChIP DNA was performed by using Genpathway's (San Diego, CA) FactorPath Discovery approach, as previously described [89]. Enriched ChIP DNA was incubated with T4 DNA polymerase to generate blunt ends, ligated to a DNA adapter X consisting of annealed 25-nt and 11-nt oligonucleotides [90], and PCR amplified using the 25-nt adapter X oligonucleotide as primer. A 40–50 bp tag library was generated from the amplified ChIP DNA by the strategy described in [90]. Amplified ChIP DNA was mixed with "random primer A" consisting of a fixed sequence A (containing a Bgl II restriction site) and a randomized 9-nt sequence at the 3' end. After denaturation of the DNA followed by chilling of the sample on ice, the annealed random primers were extended using Klenow DNA polymerase [90]. Extension products were purified on Sephacryl S-400 HR spin columns (GE Healthcare, Chalfont St. Giles, UK) and mixed with "random primer B", which differed from "random primer A" by having a different fixed sequence B (also containing a Bgl II restriction site). For second strand synthesis, denaturation, incubation with Klenow DNA polymerase and purification on Sephacryl S-400 HR was repeated as described above for first strand synthesis. The resulting DNA was amplified by PCR using primers A and B, and amplified DNA was electrophoresed on a native polyacrylamide gel (BioRad). DNA fragments of 80–90 bp were isolated, re-amplified with primers A and B and subjected to a second round of gel-purification. The DNA was then digested with restriction endonuclease Bgl II to excise the 40–50 bp tags representing genomic sequences. After gel-purification, tags were concatemerized by ligation in the presence of a limiting amount of adapter C containing a Bgl II-compatible 4-nt overhang on one end and a blunt-end on the other end. Ligation products were amplified using the adapter C sequence as PCR primers, and products containing 10–15 tags were gel-purified, cloned into the pCR2.1-TOPO vector (Invitrogen) and sequenced. A detailed diagram [see Additional file 1; Figure 5], summarizes these procedures for cloning and analysis of enriched ChIP DNA. Individual tag sequences were aligned to NCBI Rat Genome Build v3.4 using MEGABLAST v2.2.11. Tags producing low-scoring or multiple alignments were eliminated from consideration. Clusters were generated by grouping of tags mapping to within 2 kb of each other. Alignments were associated with annotated or predicted genes mapping to within 10 kb of each tag. The presence of a consensus p53 binding site was determined in each tag by querying 500 bp of bilateral genomic sequence flanking each tag using the p53MH algorithm [47]. Functional assessment of genetic elements associated with individual tags was performed using GoMiner [91] and grouped into ontological subsets for further analysis and selection. To confirm candidate p53 binding sites, PCR primers targeting a region within 200 bp of each selected alignment or cluster [See Additional file 1; Table 2] were used to measure the amount of sequence in immunoprecipitated sample by qPCR as described above.

### Quantitative reverse-transcriptase and real-time polymerase chain reactions

PC12 cells were NGF-differentiated as above and harvested at indicated intervals. Total RNA was harvested (Qiagen, Valencia, CA) according to manufacturer's protocol. RNA concentration was determined using a NanoDrop spectrophotometer (BioRad). Equal amounts of RNA sample were used for SuperScript II first-strand cDNA synthesis (Invitrogen). RT-PCR was carried out using 1 μL of cDNA per sample, HotStart master mix (SuperArray, Frederick, MD) and 0.25 μM each primer (IDT) in a GeneAmp 9700 PCR instrument (Applied Biosystems, Foster City, CA) for the indicated number of cycles. Equivalent volume of each PCR reaction was run on 2% TBE agarose gels containing ethidium bromide and photographed under UV illumination. Each target was amplified using an appropriate, empirically determined cycle number allowing gel-based visualization of samples within the exponential amplification phase of each reaction. Band intensity was measured by integration using ImageJ software [92] and normalized relative to naïve expression value.

qRT-PCR expression analysis was performed with cDNA prepared as above in reactions using the Syber Green qRT-PCR master mix (ABI) and analyzed by an ABI Model 7900 Prism instrument. All reactions were carried out in triplicate and measurements analyzed using the relative quantitation 2^-ΔΔCt ^method. The housekeeping gene GAPDH was used as an endogenous control to which sample measurements were normalized. The ROX passive reference dye was used to normalize reaction conditions across sample wells. Naïve PC12 RNA levels for each gene were used as the reference calibrator to which the NGF-treated sample RNA levels were compared. Statistical analysis of qRT-PCR data was carried out using Student's t-test for significance at p ≤ 0.05 comparing treatment to naïve gene expression levels.

## Authors' contributions

CB conceived of the study, coordinated its design, generated cell lines, participated in chromatin immunoprecipitation assays, sequence alignment/bioinformatics analyses, molecular/expression analyses and performed data/statistical analyses. PL participated in chromatin immunoprecipitation assays, fragment cloning/sequencing and sequence alignment. BAM conceived of the study and participated in its design and coordinated overall conduct of the study. All authors have read and approved the final manuscript.

## Supplementary Material

Additional File 1 Supplementary Data. A Supplementary data file is included that contains 5 figures and 4 tables. The 5 figures contain additional biological data (Figures 1, 2 and 3) and bioinformatic data (Figure 4) as well as a diagram of the ChIP cloning procedure (Figure 5). The tables contain ChIP statistics (Table 1), ChIP primers (Table 2), a list of p53-occupied regions that were non-NGF responsive (Table 3) and RT-PCR primers (Table 4). Click here for file

Additional File 2 Cluster Coordinates. A detailed data spreadsheet is included that contains cluster coordinates, chromosome number, and DNA sequences for 212 clusters and roughly 700 associated DNA sequence tags derived from p53 chromatin immunoprecipitation (ChIP) analysis of the rat genome. Click here for file
