# Supplementary material for: NGF-mediated transcriptional targets of p53 in PC12 neuronal differentiation
Source: BMC Genomics. 2007 May 31;8:139. doi: 10.1186/1471-2164-8-139 (PMC1894799; doi:10.1186/1471-2164-8-139)
Supplement: Additional File 1 — Supplementary Data. A Supplementary data file is included that contains 5 figures and 4 tables. The 5 figures contain additional biological data (Figures 1, 2 and 3) and bioinformatic data (Figure 4) as well as a diagram of the ChIP cloning procedure (Figure 5). The tables contain ChIP statistics (Table 1), ChIP primers (Table 2), a list of p53-occupied regions that were non-NGF responsive (Table 3) and RT-PCR primers (Table 4). [file 1471-2164-8-139-S1.pdf]

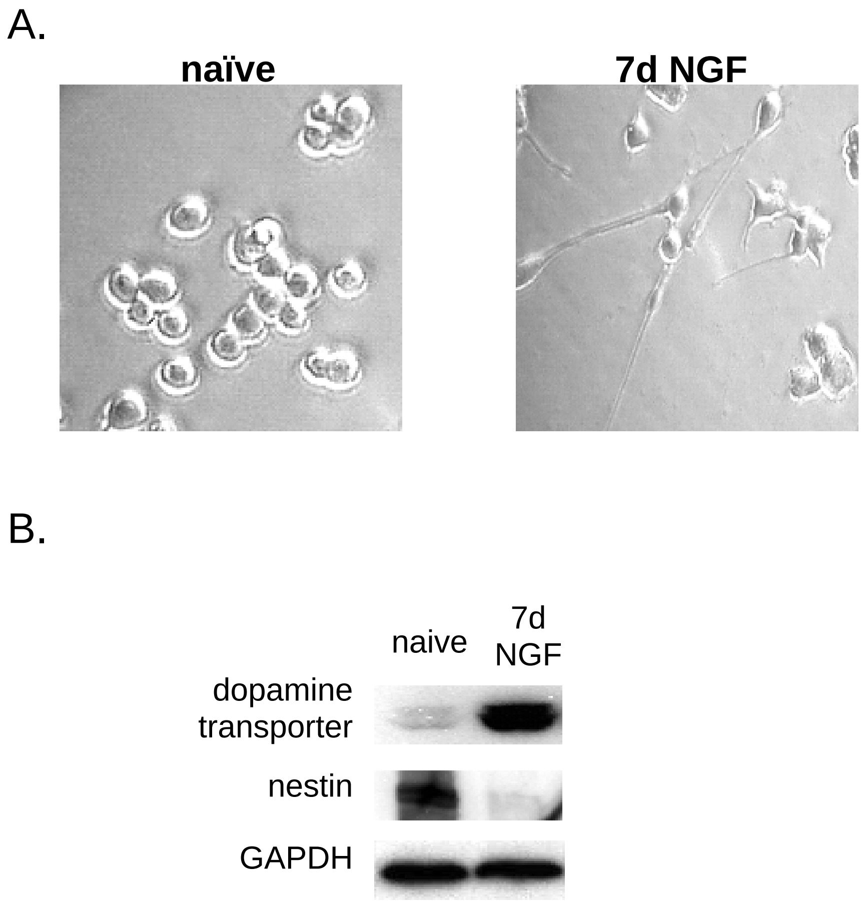

Supplementary Figure 1. PC12 cells differentiate over time following addition of NGF. A) Image at 40x magnification demonstrates neurite outgrowth after 7 days exposure to NGF. B) Western blot for indicated proteins in naïve and 7-day NGF treated PC12 cells, where GAPDH is shown as loading control.

A.

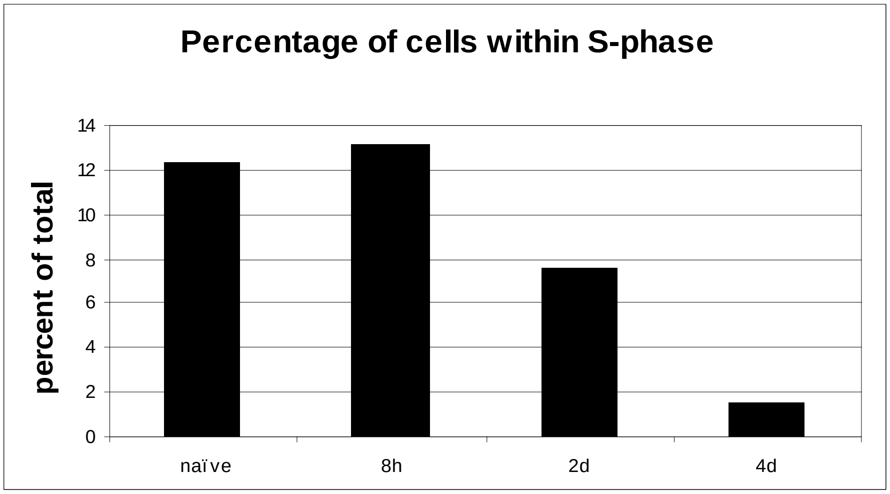

B.

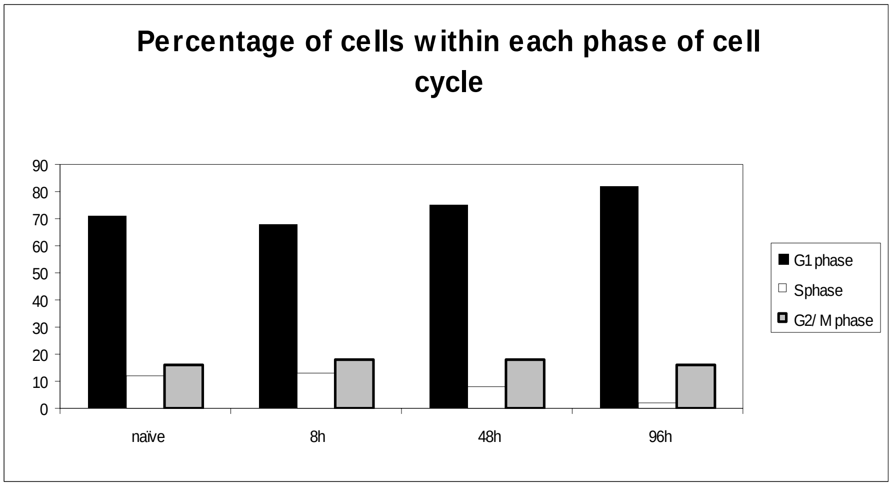

Supplementary Figure 2. NGF treatment induces G1/S cell cycle arrest. FACS cell cycle analysis was performed on NGF-treated PC12 cells at indicated time intervals using FITC-BrdU kit (Pharmingen) according to manufacturer's recommended protocol and analyzed on a Becton Dickinson FACSort flow cytometer. A) Percentage of S-phase cells, measured as proportion of cells within 10,000 recorded events. B) Percentage of cells within each phase of cell cycle, measured as percentage of cells within 10,000 recorded events.

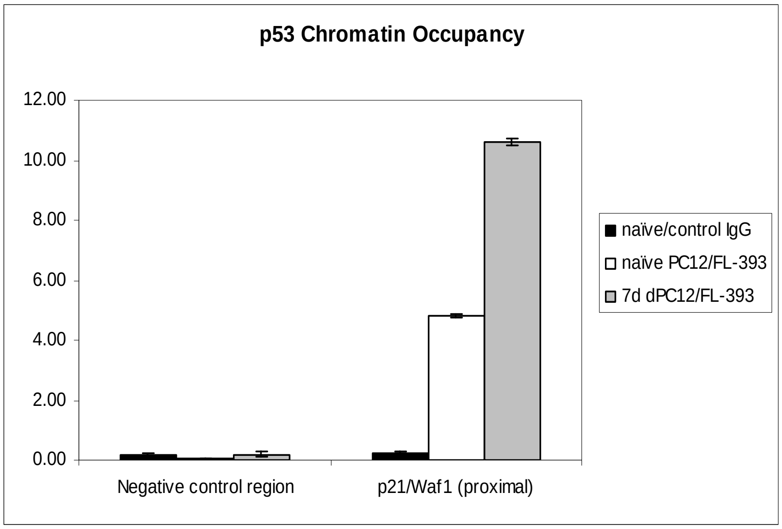

Supplementary Figure 3. NGF treatment increases p53 chromatin occupancy at the p21$^{Waf1/Cip1}$ proximal binding site. RT-PCR of ChIP-enriched DNA using either negative control IgG or anti-p53 FL-393 immunoprecipitating antibodies. Amplified regions represent a negative control genomic locus and the proximal p53 binding site within the p21$^{Waf1/Cip1}$ promoter.

A.

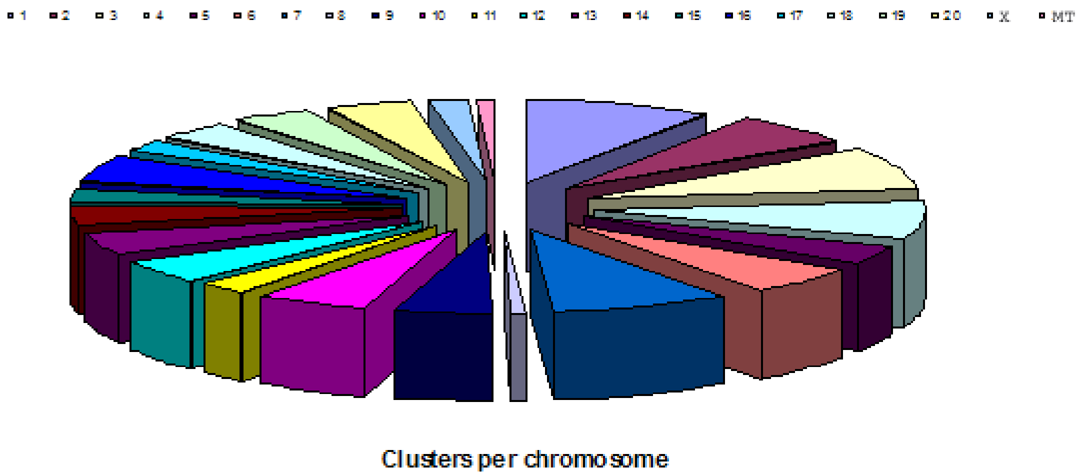

B.

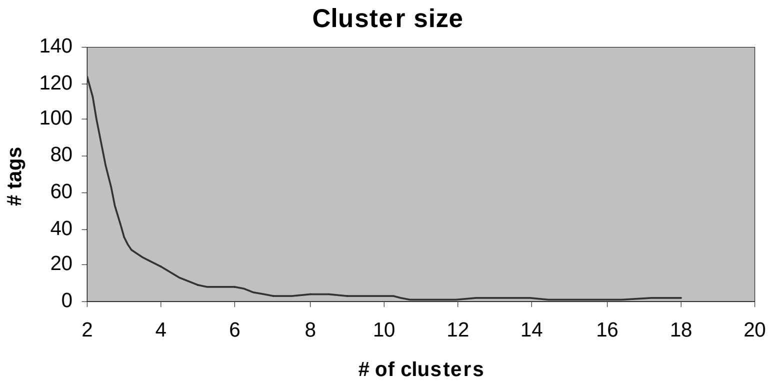

Supplementary Figure 4. Tag clusters as identified across *rattus norvegicus* genome. A) Pie chart depicting number of identified tag clusters within each chromosomal location. B) Chart depicting observed relationship between the number of tags making up each cluster and the number of clusters composed of that number of tags.

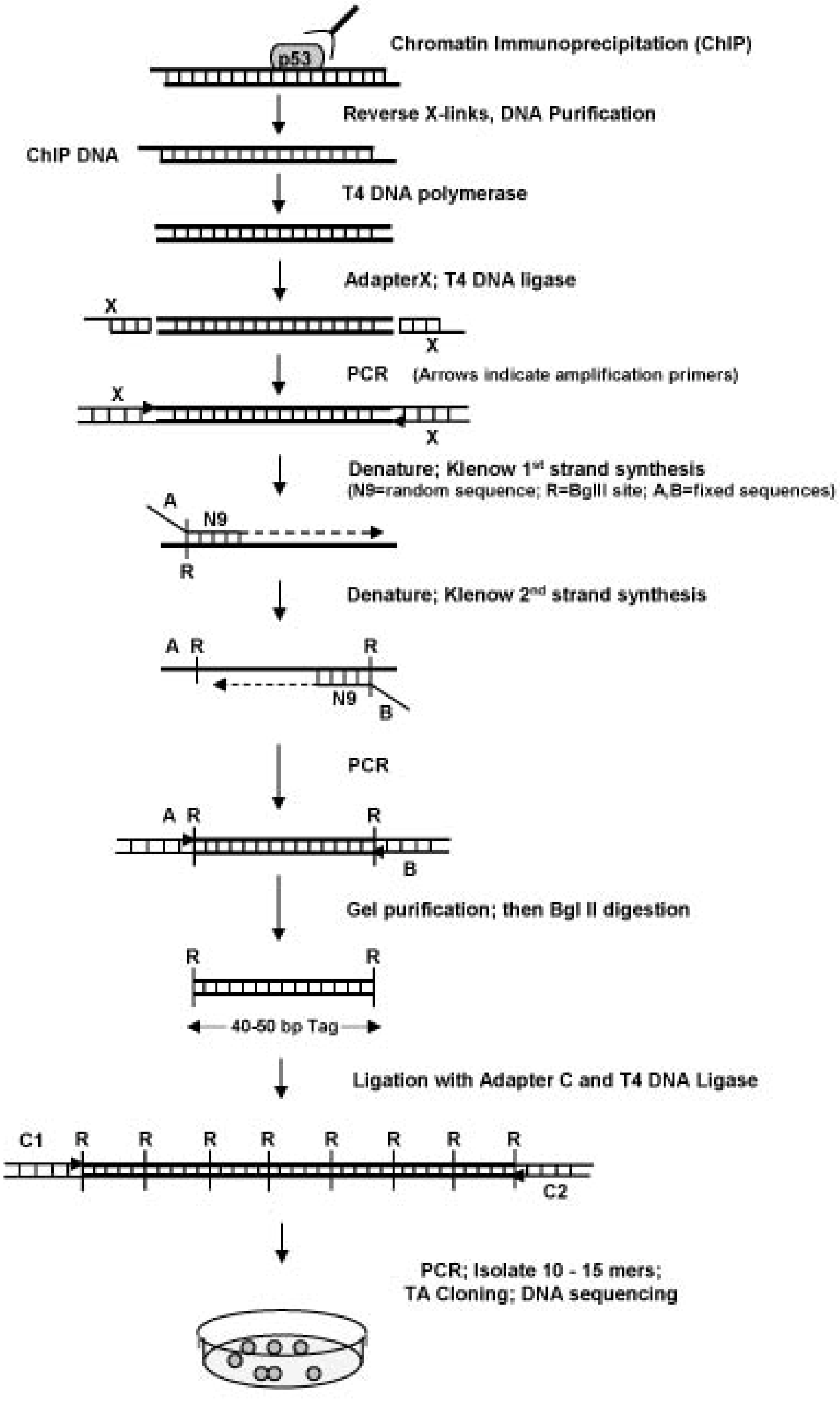

Supplementary Figure 5. Chromatin immunoprecipitation cloning procedure. Schematic outlines generation of the tag library as described in accompanying Methods text on cloning and analysis of enriched ChIP DNA. X-links = cross-links. A list of oligonucleotides used in these procedures are included below.

AdapterX 25-mer	GCGGTGACCCGGGAGATCTGAATTC
AdapterX 11-mer	GAATTCAGATC
Random primer A	CGCAGTCCTGAACCAAGATCTNNNNNNNNNN
Random primer B	TGGCATAGTGTGGCAGATCTNNNNNNNNNN
Primer A	CGCAGTCCTGAACCAA
Primer B	TGGCATAGTGTGGCAG
Adapter C1	GGCCACGCGTCGACTAGTAC
Adapter C2	*p*GATCGTACTAGTCGACGCGTGGCC

Supplementary Table 1. ChIP statistics

**Statistics**	
Number of tags	7,184
Number of alignments in Blast Output	55,911,140
Number of Blast Hits	55,911,132
Number of Hits Rejected (no contig)	0
Number of Hits Rejected (no chromosome)	1,875,389
Number of Hits better than MinBitRatio	36,970,897
Number of Hits Kept	4,770
**Tag Count**	**Distribution of Hits per Tag**
Number of tags	7,184
Number of tags with 1-3 acceptable hit(s)	3,978
Number of tags with 1 hits	3,396
Number of tags with 2 hits	372
Number of tags with 3 hits	210
Number of tags with no acceptable hit	3,206
Number of tags with 0 BLAST alignment	785
Number of tags with too many acceptable alignments	1,932
Number of tags with rejected BLAST alignments	489

Supplementary Table 2. ChIP primers

nearest gene	sequence (5' - 3')
Acvr1	CTGGGCTGCAAGGAAGACTC
	GGCTGTGTTATCAAGGCTCAC
Apc	CCTTGTATTCAGCAGGTGTACTTC
	TCCCACTCACCATTCCACTAC
Apc2	GGTTCCATCCAGCATTTATTG
	TGTGGTAAGAGGCAAGGAGTG
BRK	TCTCCCCTCTGACCACTTTG
	CCATGTGGCCAATATAGCAAG
Ccng1	CAAACAAAGAGCCCAGTCAG
	AGGTCAGAGGCAGGTGAATC
Cdc42ep4	CCTCGACCAAGAGTCCATAGAG
	CCAGGGCTTGTGTTCCTAGTC
Cnnm2	GCATTCTTGGAAGGAAGAGAAAG
	CGTAACAATAACAAAGGCACAAG
Csnk1d	GCCATCTACTGCTAGGCACAG
	GTGACCATCCCTAAACCAGAAG
Drd4	AAGCCTGCCTCTATGGACAC
	GCTGCATGTTACCCACATAAC
Ddx25	TCGCCAACTGTGTTTCTCAG
	ATGGCTCCTGCAAAGAACTC
Dlx2	AGGGGAGCAGGATCAGGTATT
	GCGCATGTCAGCTAAAGTCAT
Dusp5	AATCTCCTCCCATTCGTGTG
	AGAGGGACAGTGGGTTTGTG
Dvl3	AGAGCAAGCAGTCAGGTGTG
	GATCAGGGTGGGAGTTGAGA
Dyrk3	CCTCCCAGCTCCAAGATTAC
	CATTCAACCCTTTCCGGTAG
Ednrb	GACCCTGCAAGCAAGTCATC
	CTAGGGAGGAGGAGGAAAGTG
FAK	GAATCACACCTAATTTCCCAGTG
	GTGCTGTAGGGTTGGACTTG
Fgf2	TTACACGGCACACCTGACTG
	GGGAAAGCTGACTGGAGAAG
FLJ32743	TCAACTAACCTGTAAGCCGATTT
	ATTACTCGTGCTCTGTTATCTCAA
Gsk3b	TTCTTTGAAGCATGAAGCAGAT
	AGGCTGTCCTCCTCCTCACT
Hand1	TCTTGGCTTGATGGACGTTAG
	CTGGGTTCCTGCTACGAGAG
Hdh	GCTGACATCTGACCTGGACTC
	CGTCACCCCTGACTATTTGAC
Hipk2	TGACCATGTTAGAGGACTTGCTATT
	CACTGCCACCACCCAGTAGT
Hmmr	TTGTCCCGAGTTCCTTCTTC

	TTGAGACCCCTCGTCAGAAC
Hoxc5	GGGACTGGCACTATCCATTAC
	TAGCATCGCTTGGTCTTCTG
Kaiso	GCAGTAGGCCAGGGAAGTATG
	TCTTGCACATTTCACAACAGTG
Lect1	GCAGAACATGGGGTCTGAAG
	AGCAGCAGCAGGTGACATC
LOC300317	AGGAGGAGAGGGCTGTTATG
	CCCACAAACCCTACTTTCATC
LOC362557	GAGAAGTGTAAACAAGTAGGAAAATGAG
	CACAACCCACAGAACCTGAC
LOC366671	TCGGCAAGTGCTGTATGTTG
	CATTGGTGTGGTGATGTAGG
Mdm2	GCTTTGCCATGAGAGGGTAG
	TCGCAGTCTATTGTTGCACAG
Mdm4	TGGCTGTGTTCTTCACCATATC
	GCTGTCATTCCCAAGCTCAT
Meis1	ACGCCACTACCCTGACAAAC
	CTGTGGGATGCAAGGATTTC
MOB-like	AGATGCTGAGACACCCTGAAC
	TGCCTCTTGGAAGGATGAAC
Msh4	ACACTGCGTCCTTCACACTG
	ATCTGCCTTTGTCCCTCAAG
Nck2	TGTCTGGGCTTGTCTTCTCTC
	TTCTGTAAGGAGGACCGTGTG
Nes	CCCTTGCCTAATACCCTTGAG
	CCTCTGCATTTGGAGGACAG
Nme1	GTGCAGCTTGACAGGAACTG
	TAGAGGTGTTGCGGGAGATG
Nucb1	AGTCAGGCAGCGACATGAG
	AGGGAGGTGTGGCAGATAAC
Pkcbpb15	TGTTTTGAAGATTGGGTCTCAC
	AGGTAGGGGTGGAAGGATTAC
Pofut1	CAATGGCTCCTGGTCCTATG
	GTCCTTGCCCAGATCAGAAC
Polr3d	GTGGAGAGTCAGGCATGTTG
	CCACCGTCTCGATTTGTAGTC
Pon3	CGAGTCCTCATCACCAGTGT
	TGCAAGTTAGAGGGAATAGGAGAG
Pou2f2	TCTTATTTGGTGTCATAGCAGTGAG
	CTGCCCTAAAGGAACCAGAG
Rcc1	TGAATGTCCCAGAGCATGTG
	TGTACCCAGCATCTGCAATC
Rnf10	TTTAGGGGCCGGTGATAATG
	AGCCCTTTCTCAGGCTTGTC
Robo2(1)	TGTGAAACCCTAGAATTGGACTG
	CAAGTAAAGCTGTTTGGGTATATG
Robo2(2)	TGTGGACTTGATGGCAGATG

	AGCCTTAACGGTGGGAACAC
Rtn1	AGGAACCTGTAGTTGCTCAGTC
	CCTCCCTAGAACCAGCATTTC
Rtn4	TTGGCATTCTGTGAGCTGTG
	TTCTCTCCATGTCCTCCAAAC
Rtn4rl1	TTTCCAGCTCTGCCATACTTC
	ACCCACCCTCAAAGGTCAAG
Rtn4rl2	TCAGGAGACAGAGAGGGACAG
	TACAGGTGAGCCAGCATCTC
Sdk2	TGAACATGCCTTCCAAGTAATC
	CCTTCCTGTGTCCACCTCAG
Sesn3	AGTCAATTTAGGATTGCTCTTTATG
	ACTTGGCCTGACCCACAGAG
Shmt1	AAACCAAGCGCCACATAAAC
	CAAAACCCACGCTCCTAATAC
Slit2	TGTCGCTCTCCACTTTGTTG
	TGTCTAGTTGTGAAGGCCAGTTC
Slit3	TGGGCAGATTGGTGGAGTAG
	GGCACAGAGACTGAAGCAGAG
Snk	CATGTCCGTGAGCTTGTAGC
	CATGTGCGATTAGGGTGTTG
Tfcp2l4	GGGGAGGGCAGAGAGACTAC
	CCGGTAAACTGAGGCACAAG
Trim34	GGTGAGGGATGGAGAGAAAG
	GGCATAACTCTTGGCCTACC
Txnl2	TAGAAGCTGGGCTCTGATTG
	CGCTCACACACATAAAAGAATC
Ube2q2	TCTGGGAAGAAGGAATCTCAG
	GCAGGAACCATAGGGAACTC
usherin	CTTGGGCTGGGCTTTCTAAC
	AATGGCAATAGGACCGTGAC
Wif1	GCCCAACTAGCCATGAACTC
	CACACAATCACAGGCACTTG
Wisp1	CCATTGCCACGTCCTTAAAC
	GGAGAGAGTGGTGGGCATAA
Wnt2b	CCAGTTGGAATGCAGAGACA
	GCTGTCCAGGGAGTGAGAAG
Wnt7b	CCAGCAAGCCTCTCAAAGTG
	AGGTCAAGCTGATGGAACAAG
Znf609	CCCAGCCTAGCTTTCATTTC
	TCTCTCTTTGGATGGCAAGTC

Supplementary Table 3. p53 occupied regions; non-NGF responsive

		relative	enrichment
**target**	**Accession #**	**naïve/negative control**	**7d NGF/negative control**
Ube2q2; ubiquitin-conjugating enzyme E2Q 2	NM_031138	2	2.86
HoxC5; homeobox C5	XM_235702	5.6	5.2
Hipk2; homeodomain interacting protein kinase 2	XM_342662	5.6	2.2
Drd4; dopamine receptor D4	NM_012944	7.2	7.2
Slit3; slit homolog 3	NM_031321	6.8	7
Slit2; slit homolog 2	XM_346464	7	3.8
Hand1; heart and neural crest derivatives expressed 1	NM_021592	6.4	5.8
Rtn1; reticulon 1	NM_053865	2.8	3
Rtn4; reticulon 4	NM_031831	4	5.6
Rtn4rl1; reticulon 4 receptor-like 1	NM_181377	10.2	9.6
Rtn4rl2; reticulon 4 receptor-like 2	NM_181380	3.8	5
Robo2; roundabout homolog 2	XM_213677	3.2	1.8
Acvr1; activin A receptor, type 1	NM_053621	5.4	3.6
Ednrb; endothelin receptor type B	NM_017333	1.6	2.2
Csnk1d; casein kinase 1, delta	NM_139060	5	7
Fgf2; fibroblast growth factor 2	NM_019305	3	4.4
Nes; nestin	NM_012987	2.2	3.4
Meis1; myeloid ecotropic viral integration site 1 homolog	XM_223643	10	10.2
Dvl3; dishevelled 3, dsh homolog	XM_221304	4.2	5.8
Gsk3b; glycogen synthase kinase 3 beta	NM_032080	2.6	3.8
Wnt2b; wingless-type MMTV integration site family member 2B	XM_342308	2.6	2.2
Wisp1; WNT1 inducible signaling pathway protein 1	NM_031716	2.2	2.2
Wif1; Wnt inhibitory factor 1	NM_053738	2.4	3
Apc; adenomatosis polyposis coli	NM_012499	2.6	2.6
Apc2; adenomatosis polyposis coli 2	XM_234910	4.4	5
Pofut1; protein O-fucosyltransferase 1	NM_001002278	1.8	2.4
Hdh; Huntington disease gene homolog	XM_573634	1.4	2.2
Pou2f2; POU domain, class 2, transcription factor 2	XM_341802	4.6	6.2
Dlx2; distal-less homeobox 2	XM_230986	2	2.6
Msh4; mutS homolog 4	XM_227831	2	1.43
Cdc42ep4; Cdc42 effector protein 4	XM_221077	2.29	1.14
FAK; focal adhesion kinase	NM_013081	2.14	1.71
Cnnm2; cyclin M2	NM_001011942	2.14	2.86
Nucb1; nucleobindin 1	NM_053463	2.57	3.43
RGD1566309; similar to Kaiso protein	XM_576906	1	2
RGD1560269; similar to usherin	XM_001065756	3	2.71
Rcc1; regulator of chromatin condensation 1		2.43	2.29
Ddx25; DEAD (Asp-Glu-Ala-Asp) box polypeptide 25	NM_031630	1.57	2.14

Supplementary Table 4. gene-specific RT-PCR primers

Gene	sequence (5' - 3')
Brk	CCCACCCAACATACACAAG
	TCCCTACCCCTTCTTCTCAG
cyclin G1	ATTGCTGCCTCAATCTAGTC
	CCTGGAGTGTTTTATCAAGC
D5Ertd33E	TTCCCCTAAGGCTACAAAGC
	GCACCGACATTCTGAAGTG
Dusp5	ACCCTAGACCAAAAACCAC
	CAGACCCCAGAATCAGTAG
Dyrk3	ACCTAATGTCGGAAACCAG
	AGACATTAGCCCTTGAAGC
FLJ32743	AAAGCCCAGGAGCAAGAAC
	TCAGGCTTTCGTTTATGCG
GAPDH	ATCCCATCACCATCTTCCAG
	CCTGCTTCACCACCTTCTTG
Grnl3	AGAGCGTGGTGATGATTGTG
	TGTGCTGGACTGTGTTGAAG
Hmmr	GGAACTGTCTGCTCATCTTC
	ACTCTGCTCCTTTTTCTGC
Lect1	CCCTGACAATCCTTACCAC
	ACTCTCGTGCTAATTCTTGC
ligatin	GGAAGGTAGCAGGGAAAAGC
	GGCCAGGAAATGTCACTTG
LOC300317	AGGAGGAACTCACACAGATG
	GGGAGGGAGAAAAGGTTAG
LOC362557	TGCTCTACTCGTGGATTCAG
	GAAGTTCGGGGTGGTTTAC
LOC366671	GAACAAGCAGGAGGAAAAGG
	TGGAAGGGTCTGAAGATGG
Lrrn2	GAAGCAGCCCAGATGTCAG
	CGTCCCGTTTGGTGTAGAG
MDM2	GTGACCATTCTGCTGATTGC
	CGCTTTCTCCTGCCTGATAG
MDM4	GATTGGTATTCGGATTGCTC
	CATCGATTCCTTCATTGTCC
miR320	Ambion mirVana (sequence unknown)
MOB-like	GATGAGTGCTGTTTGTAGTG
	CAAGAAGGGGTAATAGGAG
Mpst	GAGGGTCTGGTGGATGTTC
	ACTGAGCCAGGGATGTGTC
Nck2	AGGGCAGTGAGGCTCTATG
	CCAGGGTTTCCAGTATTGC
Nme1	CATACGAGGAGACTTTTGC
	GAGGCTTCTTCCAGTTCAG
Nucdcd2	TTACGTTAGAGCGATTCCAG
	TCCACCTTTTGTGTAGTTCC
p21Waf1/Cip1	TCCTTGCCACTTCTTACCTG

	CCGGGCATCTTTGTTCTAG
p53	CAGCCAAGTCTGTTATGTGC
	GTCTTCCAGCGTGATGATG
Pkcbpb15	TTGGTAAGGGAGAAAGACTG
	GCTGAGGGGAAATAGAGTC
polR3d	GAGGATGGACAGATGGTAG
	CTGATTTGCGGATGAGTAG
Pon3	TAGCAAGGCAACCAGAAACC
	ACAAGGGGCACAAGTTCAAG
Rnf10	TCTGTGCTTTGGTTTGCTG
	TTCTTGGTTCCCTCACTTTG
Sdk2	CGAGCAGACAGGAAGCATC
	GCAATGACCACCAAGAACC
Sesn3	CCATAACTCGGCATCTGAC
	ACTGAAGCGTCAACAAACC
Shmt1	AGGGCTAGGGGTGTCATCTC
	AGGCAGGTGGAAGAGAAAGC
Snk	ACTGTGCCTCTGCTTGGATG
	GGGGAGGTTTTGGAACTGAC
Trib3	TGGTGCTAGGCTTCTGTTC
	CATCCTTTTGGCTTGAGTC
Trim34	TGTGAAGAGGGCATAGTGTC
	ATAGGCAGGTTGGGAAAAG
Txnl2	CAAAGCACCCAAGTTAGAG
	TATCCAGTCCTCCAACAAG
Wnt7b	GTCGGGCTCATGTACTACC
	GTCCTCCTCGCAGTAGTTG
Znf609	AGGAAGATGGGAAGGAAAG
	ATACGGGGAGAAAGAATAGC
